# Host-associated Intraspecific Phenotypic Variation in the Saprobic Fungus *Phlebiopsis gigantea*

**DOI:** 10.1007/s00248-023-02176-z

**Published:** 2023-01-28

**Authors:** Dārta Kļaviņa, Guglielmo Lione, Kristīne Kenigsvalde, Martina Pellicciaro, Indriķis Muižnieks, Lauma Silbauma, Jurģis Jansons, Tālis Gaitnieks, Paolo Gonthier

**Affiliations:** 1 Latvian State Forest Research Institute Silava, Rigas Street 111, Salaspils, 2169 Latvia; 2 Department of Agricultural, Forest and Food Sciences (DISAFA), University of Torino, Largo Paolo Braccini 2, 10095 Grugliasco, Italy; 3 Department of Microbiology and Biotechnology, University of Latvia, Jelgavas Street 1, Riga, 1586 Latvia

**Keywords:** Fitness, Fungi, Mycelium growth, Phenotype variability, Sporulation, Wood

## Abstract

Whether intraspecific phenotypic variation in saprobic fungi may be driven by the host of origin has received little attention. We addressed this issue by testing hypotheses using the model system *Phlebiopsis gigantea*, a wood destroying fungus associated with *Picea abies* and *Pinus sylvestris*, among others, and widely employed in practical forestry as a biological control agent. By examining approximately 60 sympatric *P. gigantea* isolates from both *P. abies* and *P. sylvestris*, we showed that the former grew in vitro significantly (*P* < 0.05) slower than the latter (average 5.56 mm/day vs. 6.84) while producing 1.8-fold significantly higher number of mitospores. An overall significant trade-off between these two phenotypic traits was detected, in particular for isolates originating from *P. abies*. Comparative inoculation experiments of a subsample of isolates and the assessment of mycelial growth in logs of both hosts allowed to reject the hypothesis that isolates are equally fit in terms of growth rate in wood of both hosts regardless of the host of origin. Tree models revealed that the growth rate of isolates was associated not only with the wood species in which the isolates were inoculated (*P* < 0.001), *P. sylvestris* being more rapidly colonized than *P. abies*, but also with the host of origin of isolates (*P* < 0.001). Results showed that *P. gigantea* isolates originating from different hosts differ phenotypically in terms of some key phenotypic traits demonstrating that a host-driven intraspecific phenotypic variation may occur in saprobic fungi.

**Supplementary Information:**

The online version contains supplementary material available at 10.1007/s00248-023-02176-z.

## Introduction

Fungi play important roles in forest ecosystems as symbionts (i.e. mycorrhizal associates), pathogens or endophytes, but a majority of them is involved in nutrient recycling as wood decomposers [1]. These are saprobic fungi either for their whole life or during specific phases of their lifecycle, as it occurs in the case of necrotrophic pathogens [2] or even endophytes [3].

While symbionts, pathogens and endophytes usually display remarkable levels of host preference, saprobes are commonly less selective [4]. A good example is that of the necrotrophic fungal species complex *Heterobasidion annosum* (Fr.) Bref. *sensu lato* (s.l.), causing destructive diseases on conifers. Although *Heterobasidion* species from Europe (*H. abietinum*, *H. annosum*, *H. parviporum*) and North America (*H. irregulare*, *H. occidentale*) mainly attack different host species [5], they are much less selective when they infect stumps by means of spores and subsequently decompose wood [6, 7].

With reference to individual fungal or fungal-like species, it was previously reported that the association with certain hosts may have an effect on some phenotypic traits, sometimes playing a role on the fitness of the microbe. Such phenomenon, known as intraspecific phenotypic variation, or phenotypic plasticity when a single genotype is involved, is well documented for symbionts, pathogens or endophytes [8–10]. Conversely, as outlined by Hapuarachchi et al. [11], examples of host-associated intraspecific phenotypic variation or phenotypic plasticity are scant in the case of saprobic fungi. Nevertheless, such phenomena might operate for saprobic fungi as well. One expectation could be that individuals originating from one host are more fit on that host than on others, as documented while assessing virulence of some plant pathogens, for example [12]. Alternatively, given the lower level of host specificity of saprobic fungi compared to pathogens, fitness on different hosts could be comparable, or even higher on hosts other than those of origin.

Only two phenotypic traits are widely recognized as metrics for the fitness of filamentous fungi: the amount of spores produced and the mycelial growth rate [13, 14]. In the case of saprobic fungi decomposing wood, these traits may be particularly relevant. Indeed, sporulation ability affects the establishment potential of the fungus. While some wood decay fungi may be already present in the living tree as pioneer microbes, many of them arrive later thanks to spore dispersal when the tree is felled [15]. The mycelial growth rate has been reported as related to the ability of the fungus to colonize and decompose wood [16]. Whether there might be synergy, independence, or trade-off between sporulation and growth in the case of saprobic fungal species decomposing wood and whether this may depend on the host are still open questions.

In this paper, we addressed the knowledge gaps outlined above by working on the generalist wood decay saprobic fungal species *Phlebiopsis gigantea* (Fr.) Jülich, a very well-known fungus being the most widely used biological control agent (BCA) worldwide in practical forestry. This BCA, commercialized under different formulations in both Europe and North America, including Rotstop^®^, is effective in preventing stump infection by *H. annosum* s.l. [5, 17]. *P. gigantea* efficacy against *H. annosum* s.l. on stumps is mainly explained by mechanisms of nutrient competition, both fungi being wood decay agents establishing on freshly cut surfaces [18, 19]. *P. gigantea* is a basidiomycete primarily found on Norway spruce (*Picea abies* (L.) Karsten) and, especially, on pine (*Pinus* spp.) stumps [20]. A recent study identified three genetically differentiated lineages within *P. gigantea*, one from Europe, one from eastern North America and one from California [21]. However, there is no information on whether intraspecific variation between sympatric populations of the fungus associated with different hosts may occur. So far, different studies have investigated the wood colonization ability of *P. gigantea* in either Sots pine (*P. sylvestris* L.) [22], or Norway spruce [23], yet few comparative reports have been published on its ability to colonize the wood of both tree species [24, 25]. One report involved the Rotstop^®^ strain originally isolated from Norway spruce in Finland, which was found to colonize Scots pine much more effectively than Norway spruce [26]. A second report involved few *P. gigantea* strains isolated from Norway spruce in Fennoscandia and from Scots pine in UK [27]. In this study, regardless of their origin, *P. gigantea* isolates were much more effective at colonizing Scots pine than Norway spruce. However, that study was not designed to test whether isolates originating from one host could be more, equally, or less efficient at colonizing that host compared to the other one. More importantly, the strains isolated from different hosts originated from distinct geographical regions; hence, the role of the host and the geographical region of origin could not be disentangled.

The aim of this study was to explore the occurrence of intraspecific phenotypic variation in sympatric populations of *P. gigantea* by testing (i) whether isolates obtained from Norway spruce and Scots pine differ in terms of mycelial growth and spore production in vitro; and (ii) whether, and to what extent, the growth rate on wood displayed by isolates may be associated with the host they originate from, with the host they colonize, or with both. Our null hypotheses were that isolates from the two hosts do not differ phenotypically in terms of mycelial growth rate and spore production in vitro and that they are equally fit in terms of growth rate on wood of both hosts, regardless of their host of origin. An additional goal was to explore whether there might be a trade-off between mycelial growth and sporulation in vitro for *P. gigantea*, and whether this can be detected on populations from both host species. In this case, our null hypothesis was that there is no trade-off between the two phenotypic traits for isolates from either host.

## Methods

### Biological Material Used in the Experiments

In 2006–2011, individual wood samples 40 × 20 × 20 mm in size were collected under *P. gigantea* fruiting bodies present in Norway spruce and Scots pine logs, stumps, or windthrown trees in 20 different sites of Latvia (Online Resource 1). For fungal isolations in the laboratory, slivers taken from wood samples were surface sterilized on the flame and put on malt extract agar (MEA) medium in Petri dishes. A total of 123 *P. gigantea* isolates, 58 from Norway spruce and 65 from Scots pine, were obtained (Online Resource 1) and used within a few weeks in the experiments described below.

### Assessment of Mycelial Growth Rates and Spore Production In Vitro

All *P. gigantea* isolates were assessed for mycelial growth as reported in Sun et al. [28] and for mitospore production as follows. Three replicates for each isolate were grown for 4 weeks on MEA medium in Petri dishes, and then gently rinsed to suspend the mitospores in one litre of tap water. Afterwards, 0.5 ml of suspension was transferred into a new Petri dish filled with MEA medium and distributed evenly on the surface. Mitospores were counted in 30 sight fields using a light microscope as described in Sun et al. [28]. For comparative purposes, the Rotstop^®^ isolate (Verdera Oy, Finland, strain code VRA 1835) was included in the experiment.

### Assessment of *P. gigantea* Growth Rate in Norway Spruce and Scots Pine Logs

The assessment of the growth rate of *P. gigantea* in wood logs was conducted by testing isolates of *P. gigantea* obtained from Norway spruce and inoculated in logs of Norway spruce or Scots pine, and isolates obtained from Scots pine inoculated in logs of Scots pine or Norway spruce. In a first inoculation trial (whole set trial), isolates of *P. gigantea* obtained from Norway spruce were inoculated in logs of Norway spruce (41 isolates) or Scots pine (17 isolates), and isolates from Scots pine were inoculated in logs of Scots pine (15 isolates) or Norway spruce (23 isolates). In a second trial (subset trial), a subset of 15 isolates obtained from Norway spruce were inoculated in both logs of Norway spruce and Scots pine, and 14 obtained from Scots pine were inoculated in both logs of Norway spruce and Scots pine. All isolates of *P. gigantea* were obtained from wood samples, as previously described. Further information on the experimental design is reported in Online Resource 2.

**Fig. 1:**
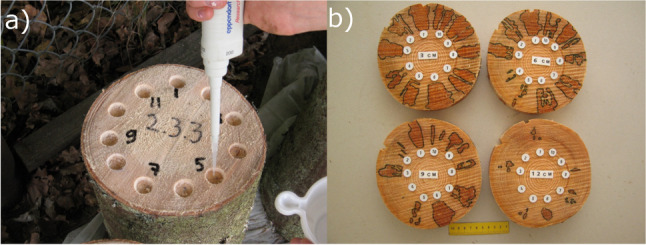
Inoculation of the spore suspension of *Phlebiopsis gigantea* in the pits drilled in wood logs (a) and areas colonized by the fungus (numbers 1 to 10) at different depths beneath the top of the logs (3, 6, 9, and 12 cm)

The experiments were conducted in the field by using the methodology described by Zaluma et al. [29]. Logs (11–17 cm diameter, 25–30 cm length) were cut from trees of comparable diameter and height, growing in a single site of Eastern Latvia (Forest Research Station Kalsnava territory, lat. 56.6889°, long. 25.9500°) characterized by uniform soil and climate conditions. For each log, 8–15 round pits 1.5 cm in diameter and 0.5 cm in depth were drilled at equal distance on the upper surface of the sapwood. The pits were filled using an automatic pipette (Fig. 1a) with 0.4 ml of *P. gigantea* spore suspension (5000 spores/ml) obtained as described above. Each isolate was inoculated in 6–33 logs. Inoculation of multiple isolates of *P. gigantea* (up to 15) was conducted in each log. After a month of incubation in field conditions and a week in the laboratory, 2–3 cm thick discs were obtained from each log. Both sides of all sample discs were examined. The presence and axial growth of *P. gigantea* in the sample discs were assessed through the observation of the typical orange-brown wood staining and of the morphological characteristics of the mycelium (Fig. 1b) [30]. To confirm the presence of *P. gigantea* in stained wood, microbial isolations were performed as described above from a subsample of 15 stained wood discs. The area occupied by staining associated with *P. gigantea* was redrawn on transparent sheets and measured with a planimeter. The depth from the top disc surface to the upper or lower side of the disc where the latest *P. gigantea* staining was identified was measured with a ruler. Growth rates (mm/day) were calculated by dividing the depth of staining associated with *P. gigantea* by the number of incubation days [29].

### Calculations and Statistics

Unbiased recursive partitioning tree model algorithms based on conditional inference were set and fitted [31–33] to compare the average in vitro mycelial growth rates (IVGR, in mm/day) and the production of mitospores (in vitro sporulation, SP, in millions per Petri dish) of *P. gigantea* obtained from Norway spruce and Scots pine. Correlation, linear equation interpolation, tree model, and standard deviational ellipse analyses were conducted to assess the relation between in vitro mycelial growth rate and sporulation [31, 32, 34, 35]. Wood growth rate of *P. gigantea* (WGR) was included as response variable in tree and random forests models [31–33, 36–38] to test its dependency upon the host of origin of the isolates, the wood species in which the isolates were inoculated, or both, and to quantify the strength of such dependency. All averages were calculated along with the 95% confidence intervals (CI_95%_) [39, 40]. Data analyses were conducted with R version 3.6.0 [41]. Additional information on data analysis is reported in Online Resource 2.

## Results

### Mycelial Growth Rates and Spore Production In Vitro

**Fig. 2:**
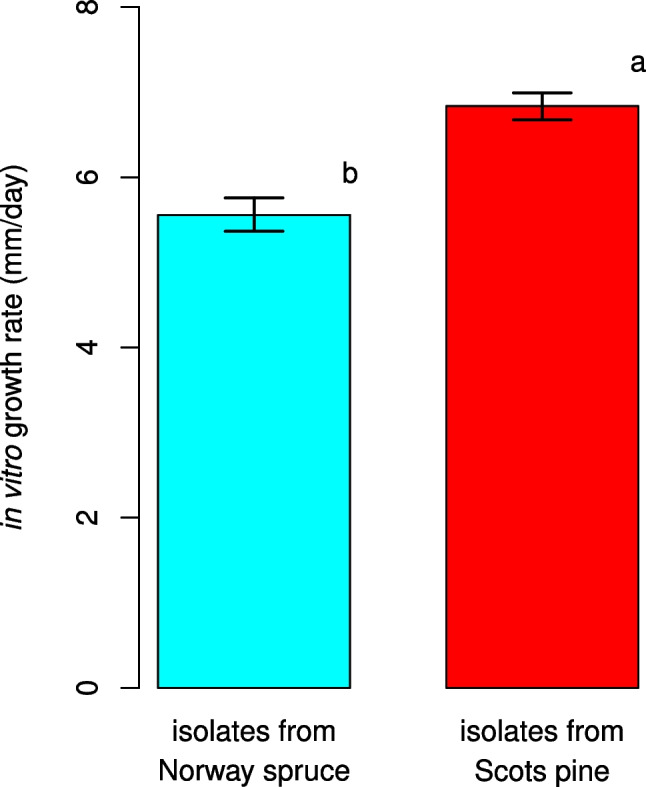
Comparison of in vitro growth rates (mm/day) of *P. gigantea* between isolates obtained from Norway spruce and Scots pine. Error bars refer to the lower and upper bounds of the 95% confidence interval. Different letters indicate significant differences (*P* < 0.05)

The analyses of the in vitro growth rates of *P. gigantea* (IVGR) showed that isolates from Scots pine grew significantly faster (6.84 mm/day, 6.68–6.99 CI_95%_) than those obtained from Norway spruce (5.56 mm/day, 5.36–5.76 CI_95%_) (*P* < 0.05) (Fig. 2).

Isolates of *P. gigantea* were split by the tree model among 12 clusters with significantly different averages (*P* < 0.05) (Online Resource 3). Isolates from Scots pine were predominant (57–89%) in 5 of the clusters displaying the highest average IVGR (6.47–9.21 mm/day), while the abundance of isolates from Norway spruce was prevalent (53–100%) in the other clusters, whose IVGR mean values were significantly lower (2.80–5.90 mm/day) (*P* < 0.05) (Online Resource 3). The Rotstop^®^ strain of *P. gigantea* displayed an average IVGR of 6.69 mm/day (6.24–7.23 CI_95%_).

**Fig. 3:**
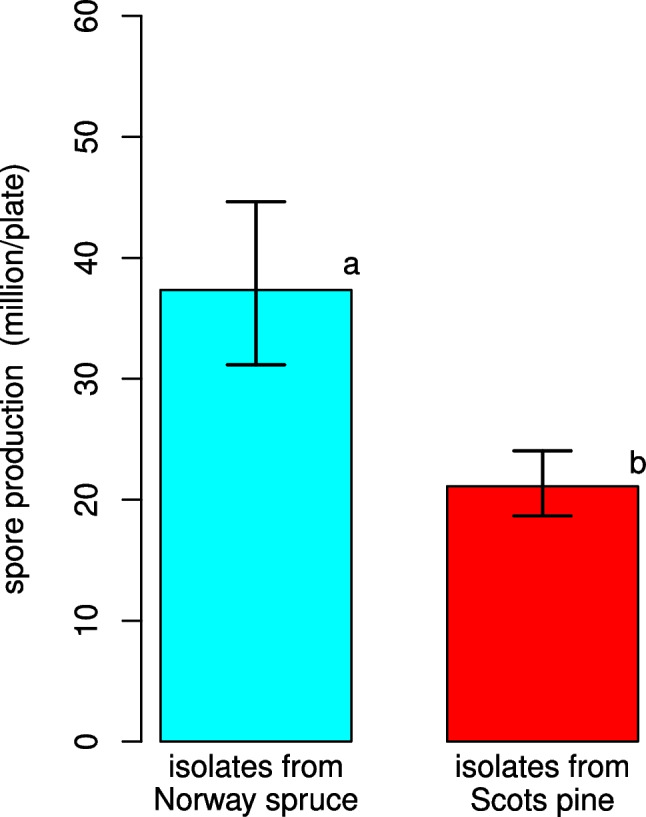
Comparison of in vitro sporulation (mm/day) of *Phlebiopsis gigantea* between isolates obtained from Norway spruce and Scots pine. Error bars refer to the lower and upper bounds of the 95% confidence interval. Different letters indicate significant differences (*P* < 0.05)

When analyzing the in vitro sporulation of *P. gigantea*, isolates from Norway spruce scored 1.8-fold higher (37.35 million/plate, 31.15–44.62 CI_95%_) than those from Scots pine (21.11 million/plate, 18.67–24.04 CI_95%_) (*P* < 0.05) (Fig. 3).

The tree model outlined the presence of 9 clusters characterized by significant differences (*P* < 0.05) among the average SP values (Online Resource 4). Clusters with higher SP values (45–160 million/plate) included a large majority of isolates from Norway spruce (55–100%), while most of the clusters displaying lower SP values (1–32 million/plate) were mainly composed by isolates from Scots pine (up to 75%) (Online Resource 4). The Rotstop^®^ strain of *P. gigantea* displayed an average SP of 35.40 million/plate (20.02–51.82 CI_95%_).

### Trade-off Between Mycelial Growth Rate and Spore Production In Vitro

**Fig. 4:**
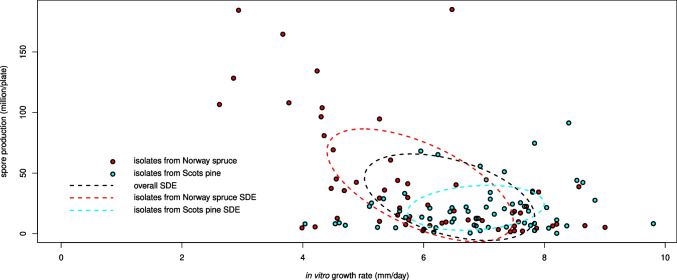
Correlation analysis between in vitro growth rate and spore production of *P. gigantea* isolates. The scatterplot shows for each host tree species (Norway spruce and Scots pine) from which isolates were obtained the growth rate in vitro (*x*-axis, in mm/day) and the corresponding spore production (*y*-axis, in million/plate). Correlations between the two variables are represented as standard deviational ellipses (SDE) fitted on the total number of isolates (overall SDE), and separately for the two host tree species (isolates from Norway spruce SDE and isolates from Scots pine SDE)

The correlation analysis between in vitro growth rate and sporulation indicated an overall trade-off between the average IVGR and SP values of *P. gigantea* isolates (Rho =  − 0.301, *P* < 0.01) (Fig. 4). The slope coefficient (*m*) or the linear equation interpolating IVGR and SP values attained − 11.394 $\frac{{~}^{million\; spores}\!\left/ \!{~}_{plate}\right.}{{~}_{mm\; mycelial\; growth}\!\left/ \!{~}_{day}\right.}$ (*P* < 0.05), corresponding to an average reduction of approximately 11 million spores per Petri plate for each additional increase of 1 mm/day of the mycelial growth rate. A similar trade-off was observed for the isolates from Norway spruce (Rho =  − 0.608, *P* < 0.05; *m* =  − 18.430 $\frac{{~}^{million\; spores}\!\left/ \!{~}_{plate}\right.}{{~}^{mm\; mycelial\; growth}\!\left/ \!{~}_{day}\right.}$, *P* < 0.05), but not for those from Scots pine (Rho = 0.131, *P* > 0.05; *m* =  − 2.914 $\frac{{~}^{million\; spores}\!\left/ \!{~}_{plate}\right.}{{~}^{mm\; mycelial\; growth}\!\left/ \!{~}_{day}\right.}$, *P* > 0.05). The standard deviational ellipse orientations were consistent with the results of the correlation analyses (Fig. 4).

The tree model fitted to the overall data detected that isolates of *P. gigantea* with a growth rate over 4.35 mm/day resulted in an average spore production fourfold lower than that of the other isolates (23 vs. 94 million spores/plate, *P* < 0.05). A similar result was obtained for the isolates from Norway spruce, with thresholds at 4.35 and 6.53 mm/day corresponding to a reduction from 101 to 34 and from 34 to 11 million spores/plate (*P* < 0.05). No significant thresholds (*P* > 0.05) were detected for isolates from Scots pine.

### Growth Rate of *P. gigantea* Isolates Originated from Different Hosts in Norway Spruce and Scots Pine Logs

Tree model algorithms showed that the growth rate of the isolates of *P. gigantea* (i.e. WGR) was significantly associated with both the host of origin of the isolates (i.e. host species from which the isolates were obtained) (*P* < 0.001), and with the wood species in which the isolates were inoculated (*P* < 0.001). Nonetheless, WGR was mostly dependent upon the host in which the isolates were inoculated, rather than on the host of origin. In fact, the former resulted in the highest values of variable importance (0.533 for the whole set of data, 0.461 for the subset) and in the lowest Theil’s UII coefficients (0.409 and 0.427). Conversely, the host of origin displayed the lowest variable importance (0.254 for the whole dataset, 0.390 for the subset) and the highest UII coefficient (0.431 and 0.433). In addition, the most substantial differences between averages of WGR were associated with the hosts in which isolates were inoculated, rather than with the hosts of origin, but with opposite trends between Norway spruce and Scots pine. In fact, single-predictor tree models showed that isolates inoculated in Scots pine significantly outgrew (*P* < 0.05) those inoculated in Norway spruce with an average WGR of 2.95 mm/day (2.85–3.05 CI_95%_) vs. 2.02 (1.98–2.01 CI_95%_) (i.e. + 0.93 mm/day) for the whole dataset, and 2.86 mm/day (2.76–2.97 CI_95%_) vs. 2.01 mm/day (1.93–2.09 CI_95%_) (i.e. + 0.85 mm/day) for the subset. On the other side, isolates originating from Norway spruce were significantly faster growing (*P* < 0.05) than those obtained from Scots pine with an average WGR of 2.50 mm/day (2.45–2.56 CI_95%_) vs. 1.98 (1.91–2.05 CI_95%_) (i.e. + 0.52 mm/day) for the whole dataset, and 2.84 mm/day (2.74–2.96 CI_95%_) vs. 2.08 mm/day (1.99–2.16 CI_95%_) (+ 0.76 mm/day) for the subset.

**Fig. 5:**
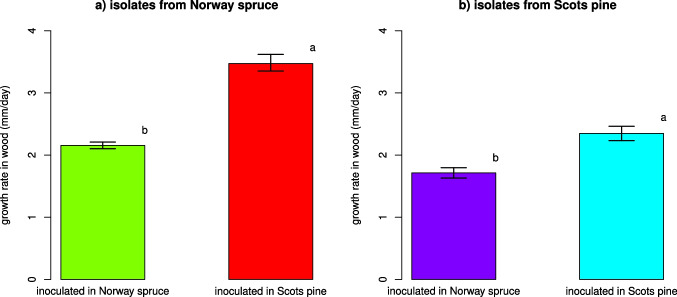
Comparison of growth rates (mm/day) of *P. gigantea* isolates from Norway spruce (**a**) and Scots pine (**b**) inoculated in wood samples of both host species. Error bars refer to the lower and upper bounds of the 95% confidence interval. Different letters mark significant differences (*P* < 0.05)

Models including the whole dataset and both host types (i.e. host of origin and inoculated host) showed that the strains from Norway spruce inoculated in Scots pine were the best wood colonizers (considering the linear colonization rate as a proxy to assess the wood colonization ability) with an average WGR of 3.47 mm/day (3.35–3.61 CI_95%_), followed by those obtained from Scots pine and inoculated in Scots pine (2.35 mm/day, 2.23–2.46 CI_95%_) and by those from Norway spruce inoculated in Norway spruce (2.15 mm/day, 2.10–2.21 CI_95%_), while isolates from Scots pine grown in Norway spruce displayed the lowest growth rate (1.71 mm/day, 1.63–1.80 CI_95%_) (Fig. 5).

Comparable results were obtained from the analysis of the data subset (Online Resource 5). All the above averages differed significantly (*P* < 0.05). The Rotstop^®^ strain of *P. gigantea* displayed an average WGR of 1.53 mm/day (1.41–1.65 CI_95%_) on Norway spruce and 2.35 mm/day (2.15–2.56 CI_95%_) on Scots pine.

## Discussion

All lines of evidence point to the occurrence of intraspecific phenotypic variation in the saprobic fungus *P. gigantea*, at least for the investigated traits. As intraspecific phenotypic variation could be due to other factors in addition to the host of origin of isolates, including the geographical region and the environmental conditions in the area of origin, we minimized the risk of computing other potential influencing factors by using isolates collected within a defined geographic area (between latitudes 56° and 57°N) of a single country, i.e. Latvia. Therefore, the intraspecific phenotypic variation observed in this work in populations of *P. gigantea* should have been virtually driven by the host of origin. As the genetic characterization of isolates was out of the aims of the work, whether the observed phenotypic variation is reflected by certain levels of genetic variation remains an open question. It should be noted that European populations of *P. gigantea* are characterized by a low genetic differentiation [42, 43], hence, intra-population genetic variation among the isolates analysed in our study should have played a minor role, if any, on the observed phenotypic variation. Nevertheless, different isolates should represent different genotypes as expected based on the knowledge on the sexuality of *P. gigantea* [20].

Our in vitro experiments clearly showed that *P. gigantea* isolates originating from different hosts differed significantly at least in two relevant phenotypic traits, i.e. growth rate and spore production, supporting the hypothesis that host association may drive the phenotype of fungi. While evidence supporting this hypothesis was previously provided for fungal plant pathogens with reference to virulence [12], and for fungal symbionts with reference to growth rate and production of different structures [10], such evidence was still lacking for saprobic fungi, which are notoriously less intimately associated with host plants compared to pathogens and symbionts.

On average, *P. gigantea* isolates from Scots pine included in this study grew faster than those obtained from Norway spruce while the latter, on average, produced a larger amount of spores compared to the former. Therefore, for both traits, we reject the null hypothesis that isolates from the two hosts do not differ phenotypically. Statistical analyses indicated an overall trade-off between the two variables, i.e. IVGR and SP, at the species level, supporting previous studies on *Bionectria ochroleuca* which, again, has been reported as a fungal plant pathogen, an endophyte in plant tissues or as a free-living form rather than a strict saprobe [44]. However, statistical analyses conducted separately on data of either *P. gigantea* from Norway spruce or from Scots pine pointed to a clear and significant trade-off between the two variables only with reference to isolates collected from Norway spruce. Therefore, the null hypothesis on the lack of trade-off between the two phenotypic traits is also in principle rejected, although this might hold true for populations of *P. gigantea* associated with Scots pine. The reasons for such an asymmetric pattern depending on the host is unknown, but we believe it is unlikely to be associated with either the statistical tests employed or the unbalanced number of isolates from each host species. In fact, not only our data came from a large number of isolates from both Norway spruce and Scots pine, but the models used in this work have already proved to be robust and adequate to deal with complex experimental designs and data structures [31–33, 38].

A picture of what can happen in nature can be gathered by interpreting the results of the experiment assessing the growth rate of *P. gigantea* isolates on wood logs of both Norway spruce and Scots pine. It should be noted that our experimental design consisting of the inoculation of multiple isolates (up to 15) in each log/tree genotype should have overcome the implicit bias deriving from the inoculation of one isolate in each log/tree genotype, an approach commonly employed when inoculating wood decay fungi in wood [26, 45]. Our experimental design should have minimized the possible effects of using different host genotypes and logs characterized by different levels of moisture content, which may affect mycelial growth as demonstrated for other wood decay fungi [46]. Moreover, since the logs used for the experiment were collected from trees similar in diameter and height, and growing under homogeneous environmental conditions, we deem that wood properties (e.g. density) were rather constant and could have unlikely influenced the growth rate of *P. gigantea*.

Results of experiments conducted by using wood logs allow us to reject the null hypothesis that isolates from the two hosts are equally fit in terms of growth rate in wood of both host species. Tree models revealed that the growth rate of isolates was associated not only with the wood species in which the isolates were inoculated, Scots pine being more rapidly colonized than Norway spruce, but also with the host of origin of isolates. In fact, regardless of the origin of isolates, a faster development of *P. gigantea* in wood of Scots pine compared to wood of Norway spruce was observed in our study, supporting previously published reports [26, 27]. Interestingly, isolates from Norway spruce grew significantly faster in wood of Scots pine rather than in wood of Norway spruce. It should be noted that while the origin of the isolates is significant and plays a role in accounting for the variability observed in the wood colonization ability (i.e. growth rate), the inoculated host explains most of such variability. Therefore, we propose that the higher growth rate of *P. gigantea* observed in wood of Scots pine than in Norway spruce could be mostly related with the host species in which the isolate was inoculated rather than with the host species of origin of the isolates. Consistently with the other isolates of *P. gigantea* from Norway spruce, the Rotstop^®^ strain grew more efficiently in Scots pine than in Norway spruce, supporting previous findings [26, 27]. This may provide a clue to interpret the high effectiveness of Rotstop^®^ against *H. annosum* s.l. on pine stumps [47, 48].

Host-associated intraspecific phenotypic variation observed in *P. gigantea* may be due to different factors. For instance, chemical compounds produced by the host and epigenetic factors might influence the expression of some phenotypic traits, as previously reported for fungal species of the genera *Helminthosporium* [49] and *Nodulisporium* [50]*.* Interestingly, in both these reports [49, 50], the biosynthesis of some metabolites was substantially attenuated when the fungal strains were separated from their host substrates and grown on a different medium, suggesting a fungus-substrate interaction driving the expression of the phenotypic trait investigated (i.e. the metabolite biosynthesis). Whether, in the case of *P. gigantea*, the observed phenotypic differences depend on specific physical, biochemical, or chemical characteristics of the host wood remains an open question.

In conclusion, by performing different experiments using a large number of sympatric isolates of a well-known wood decay fungal species, we were able to show that isolates originating from different hosts differ phenotypically in terms of some key phenotypic traits. This is one of the first reports on the occurrence of a host-driven intraspecific phenotypic variation for saprobic fungi. Given the model system employed, this study could have some practical implications. For instance, the screening of potential BCA against plant pathogens of a given host generally occurs on isolates from that host. Our results suggest that the screening could profitably encompass isolates from different hosts as the latter could be even more performant because of the phenotypic variation.

## Supplementary Information

Below is the link to the electronic supplementary material.

Supplementary file1. Online Resource 1 Map of Latvian sampling sites and table reporting for each isolate of *Phlebiopsis gigantea* the corresponding code, the host species from which the isolate was obtained, and the sampling site of provenance (PDF 664 KB)

Supplementary file2. Online Resource 2 Data analysis, calculation, statistics, modelling, and software technical information (PDF 875 KB)

Supplementary file3. Online Resource 3 Clustering of the isolates of *Phlebiopsis gigantea* based on their in vitro growth rate (PDF 576 KB)

Supplementary file4. Online Resource 4 Clustering of the isolates of *Phlebiopsis gigantea* based on their in vitro sporulation (PDF 565 KB)

Supplementary file5. Online Resource 5 Comparison of growth rates (mm/day) of *Phlebiopsis gigante*a isolates from Norway spruce and Scots pine inoculated in wood samples of both host species referring to the data subset (PDF 512 KB)

## Data Availability

The datasets generated during and/or analysed during the current study are available from the corresponding author on request.
