# Supplementary material for: Host-associated Intraspecific Phenotypic Variation in the Saprobic Fungus Phlebiopsis gigantea
Source: Microb Ecol. 2023 Jan 28;86(3):1847–55. doi: 10.1007/s00248-023-02176-z (PMC10497652; doi:10.1007/s00248-023-02176-z)
Supplement: Supplementary file 1 — Supplementary file1. Online Resource 1 Map of Latvian sampling sites and table reporting for each isolate of Phlebiopsis gigantea the corresponding code, the host species from which the isolate was obtained, and the sampling site of provenance (PDF 664 KB) [file 248_2023_2176_MOESM1_ESM.pdf]

MICROBIAL ECOLOGY

Host-associated intraspecific phenotypic variation in the saprobic fungus *Phlebiopsis gigantea*

Dārta Kļaviņa [1], Guglielmo Lione [2]*, Kristīne Kenigsvalde [1], Martina Pellicciaro [2], Indriķis Muižnieks [3], Lauma Silbauma [1], Jurģis Jansons [1], Tālis Gaitnieks [1] and Paolo Gonthier [2]

[1] Latvian State Forest Research Institute Silava, Rigas street 111, LV-2169, Salaspils, Latvia.

[2] Department of Agricultural, Forest and Food Sciences (DISAFA), University of Torino, Largo Paolo Braccini 2, I-10095, Grugliasco, Italy.

[3] Department of Microbiology and Biotechnology, University of Latvia, Jelgavas street 1, LV- 1586, Riga, Latvia.

*Corresponding author: Guglielmo Lione (email: guglielmo.lione@unito.it)

ONLINE RESOURCE 1

Map of Latvian sampling sites, table of sampling site coordinates, and table reporting for each isolate of *Phlebiopsis gigantea* the corresponding code, the host species from which the isolate was obtained, and the sampling site of provenance.

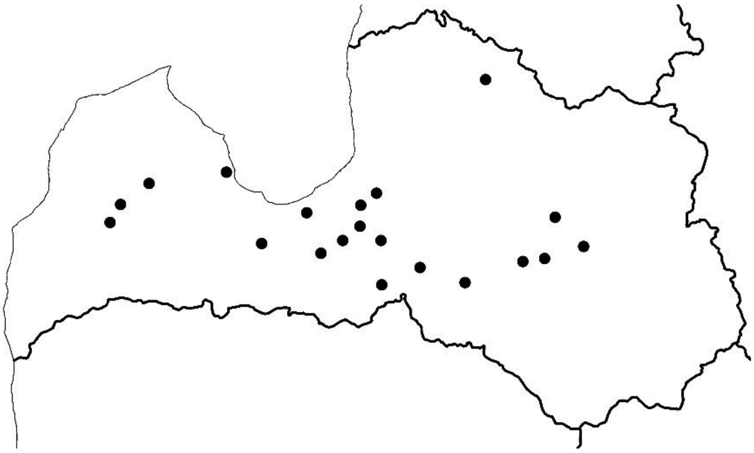

Sampling site	Latitude	Longitude
Babīte	56°57'33.1"N	23°56'49.2"E
Bārbele	56°27'12.8"N	24°35'47.5"E
Cesvaine	56°57'56.5"N	26°18'39.5"E
Daube	56°54'12.5"N	24°25'16.4"E
Druviņas	56°45'27.2"N	24°43'08.7"E
Engure	57°09'50.0"N	23°13'45.6"E
Ģibuļi	57°12'21.5"N	22°26'01.5"E
Inčukalns	57°06'07.5"N	24°41'17.9"E
Kalsnava	56°41'27.4"N	25°58'11.4"E
Kandava	57°02'19.5"N	22°46'42.2"E
Kuldīga	56°58'40.7"N	21°57'52.8"E
Lemzeri	57°04'60.0"N	22°04'00.0"E
Mētriena	56°40'47.4"N	26°19'27.9"E
Ogre	56°48'57.0"N	24°36'21.8"E
Ozoli	56°59'04.2"N	26°03'26.9"E
Plakanciems	56°46'05.9"N	24°06'32.4"E
Salaspils	56°52'11.9"N	24°20'50.7"E
Skrīveri	56°38'35.2"N	25°06'56.1"E

Sampling site	Latitude	Longitude
Strenči	57°37'50.6"N	25°41'28.6"E
Tīreļi	56°50'43.9"N	23°35'15.7"E

**Isolate of *P. gignatea***	**Host species from which the isolate was obtained**	**Sampling site**	**Isolate of *P. gignatea***	**Host species from which the isolate was obtained**	**Sampling site**
B107E	*P. abies*	Bārbele	D107P	*P. sylvestris*	Druviņas
B207E	*P. abies*	Bārbele	E107P	*P. sylvestris*	Engure
B307E	*P. abies*	Bārbele	Gi107P	*P. sylvestris*	Ģibuļi
B407E	*P. abies*	Bārbele	Gi207P	*P. sylvestris*	Ģibuļi
B507E	*P. abies*	Bārbele	Gi307P	*P. sylvestris*	Ģibuļi
B607E	*P. abies*	Bārbele	Ģ20110P	*P. sylvestris*	Ģibuļi
B707E	*P. abies*	Bārbele	Ģ20210P	*P. sylvestris*	Ģibuļi
In208E	*P. abies*	Inčukalns	Ģ20310P	*P. sylvestris*	Ģibuļi
K0111E	*P. abies*	Kalsnava	Ģ20510P	*P. sylvestris*	Ģibuļi
K0211E	*P. abies*	Kalsnava	Ģ20810P	*P. sylvestris*	Ģibuļi
K1	*P. abies*	Kalsnava	In108P	*P. sylvestris*	Inčukalns
K2	*P. abies*	Kalsnava	J1	*P. sylvestris*	Daube
K3	*P. abies*	Kalsnava	J1007P	*P. sylvestris*	Daube
K4	*P. abies*	Kalsnava	J107P	*P. sylvestris*	Daube
Kd107E	*P. abies*	Kuldīga	J1107P	*P. sylvestris*	Daube
Kd308E	*P. abies*	Kuldīga	J1207P	*P. sylvestris*	Daube
Kn107E	*P. abies*	Kandava	J1307P	*P. sylvestris*	Daube
Le107E	*P. abies*	Lemzeri	J1407P	*P. sylvestris*	Daube
M108E	*P. abies*	Mētriena	J1507P	*P. sylvestris*	Daube
M208E	*P. abies*	Mētriena	J1607P	*P. sylvestris*	Daube
NA0110E	*P. abies*	Ozoli	J1707P	*P. sylvestris*	Daube
NA0210E	*P. abies*	Ozoli	J2	*P. sylvestris*	Daube
NC0110E	*P. abies*	Ozoli	J207P	*P. sylvestris*	Daube
NC0210E	*P. abies*	Ozoli	J3	*P. sylvestris*	Daube
O1	*P. abies*	Ogre	J307P	*P. sylvestris*	Daube
O107E	*P. abies*	Ogre	J4	*P. sylvestris*	Daube
O108E	*P. abies*	Ogre	J407P	*P. sylvestris*	Daube
O2	*P. abies*	Ogre	J507P	*P. sylvestris*	Daube
O207E	*P. abies*	Ogre	J607P	*P. sylvestris*	Daube
O208E	*P. abies*	Ogre	J707P	*P. sylvestris*	Daube
Og0111E	*P. abies*	Ogre	J807P	*P. sylvestris*	Daube
Og0211E	*P. abies*	Ogre	J907P	*P. sylvestris*	Daube
Og0311E	*P. abies*	Ogre	K107P	*P. sylvestris*	Kalsnava
Og0411E	*P. abies*	Ogre	K108P	*P. sylvestris*	Kalsnava

Isolate of *P. gignatea*	Host species from which the isolate was obtained	Sampling site
Pl	*P. abies*	Plakanciems
Sk107E	*P. abies*	Skrīveri
T107E	*P. abies*	Tīreļi
T207E	*P. abies*	Tīreļi
Ti108E	*P. abies*	Kalsnava
Ti208E	*P. abies*	Kalsnava
Ti408E	*P. abies*	Kalsnava
Ti508E	*P. abies*	Kalsnava
Ti608E	*P. abies*	Kalsnava
Z0111E	*P. abies*	Kalsnava
Z0211E	*P. abies*	Kalsnava
Z0311E	*P. abies*	Kalsnava
Z0411E	*P. abies*	Kalsnava
Z0511E	*P. abies*	Kalsnava
Z0611E	*P. abies*	Kalsnava
Z0711E	*P. abies*	Kalsnava
Z0811E	*P. abies*	Kalsnava
Z0911E	*P. abies*	Kalsnava
Z1011E	*P. abies*	Kalsnava
Z1111E	*P. abies*	Kalsnava
Z1211E	*P. abies*	Kalsnava
Z1311E	*P. abies*	Kalsnava
Z1411E	*P. abies*	Kalsnava
Z1511E	*P. abies*	Kalsnava
Ap0111P	*P. sylvestris*	Salaspils
Ba0111P	*P. sylvestris*	Babīte
C1	*P. sylvestris*	Cesvaine
C2	*P. sylvestris*	Cesvaine

Isolate of *P. gignatea*	Host species from which the isolate was obtained	Sampling site
K207P	*P. sylvestris*	Kalsnava
K208P	*P. sylvestris*	Kalsnava
K307P	*P. sylvestris*	Kalsnava
K407P	*P. sylvestris*	Kalsnava
Kd1	*P. sylvestris*	Kuldīga
Kd108P	*P. sylvestris*	Kuldīga
Kd2	*P. sylvestris*	Kuldīga
Kd207P	*P. sylvestris*	Kuldīga
Kd208P	*P. sylvestris*	Kuldīga
Kd3	*P. sylvestris*	Kuldīga
Kd408P	*P. sylvestris*	Kuldīga
Kd508P	*P. sylvestris*	Kuldīga
Kd608P	*P. sylvestris*	Kuldīga
Kn1	*P. sylvestris*	Kandava
Kn207P	*P. sylvestris*	Kandava
L108P	*P. sylvestris*	Strenči
Le207P	*P. sylvestris*	Lemzeri
Le307P	*P. sylvestris*	Lemzeri
Le407P	*P. sylvestris*	Lemzeri
Le507P	*P. sylvestris*	Lemzeri
Le607P	*P. sylvestris*	Lemzeri
Le707P	*P. sylvestris*	Lemzeri
Le807P	*P. sylvestris*	Lemzeri
N107P	*P. sylvestris*	Ozoli
N207P	*P. sylvestris*	Ozoli
S107P	*P. sylvestris*	Salaspils
S207P	*P. sylvestris*	Salaspils
