# Supplementary material for: Host-associated Intraspecific Phenotypic Variation in the Saprobic Fungus Phlebiopsis gigantea
Source: Microb Ecol. 2023 Jan 28;86(3):1847–55. doi: 10.1007/s00248-023-02176-z (PMC10497652; doi:10.1007/s00248-023-02176-z)
Supplement: Supplementary file 2 — Supplementary file2. Online Resource 2 Data analysis, calculation, statistics, modelling, and software technical information (PDF 875 KB) [file 248_2023_2176_MOESM2_ESM.pdf]

MICROBIAL ECOLOGY

Host-associated intraspecific phenotypic variation in the saprobic fungus *Phlebiopsis gigantea*

Dārta Kļaviņa [1], Guglielmo Lione [2]*, Kristīne Kenigsvalde [1], Martina Pellicciaro [2], Indriķis Muižnieks [3], Lauma Silbauma [1], Jurģis Jansons [1], Tālis Gaitnieks [1] and Paolo Gonthier [2]

[1] Latvian State Forest Research Institute Silava, Rigas street 111, LV-2169, Salaspils, Latvia.

[2] Department of Agricultural, Forest and Food Sciences (DISAFA), University of Torino, Largo Paolo Braccini 2, I-10095, Grugliasco, Italy.

[3] Department of Microbiology and Biotechnology, University of Latvia, Jelgavas street 1, LV- 1586, Riga, Latvia.

*Corresponding author: Guglielmo Lione (email: guglielmo.lione@unito.it)

ONLINE RESOURCE 2

Data analysis, calculation, statistics, modelling, and software technical information

The average *in vitro* mycelial growth rates (IVGR, in mm/day) of *Phlebiopsis gigantea* were compared between isolates obtained from Norway spruce (58 isolates with a total of 249 replicates, i.e. fungal colonies in Petri dishes) and Scots pine (65 isolates, 201 replicates) by fitting an unbiased recursive partitioning tree model based on conditional inference (Hothorn *et al.* 2006, Hothorn and Zeileis 2015). The algorithm used to fit the model was set as described in Lione *et al.* (2020), with IVGR used as output variable and the host tree species as input categorical variable.

A second unbiased recursive partitioning tree model was fitted to test if *P. gigantea* isolates could be split in different clusters based on their IVGR. Clusters were defined by identifying the sets of *P. gigantea* isolates partitioned among the terminal nodes of the tree model, as resulting at the end of the binary split process at *P*-value $P<0.05$ (Hothorn *et al.* 2006, Hothorn and Zeileis 2015). The average IVGR of the isolates included in each cluster was calculated along with the ratio (%) between the numbers of isolates obtained from Norway spruce and Scots pine and the total number of isolates partitioned within the same cluster.

The production of mitospores *in vitro* (hereafter referred to as sporulation, SP, in millions per Petri dish) was analyzed as described for IVGR on the same isolates, for which 188 and 209 replicates were available for isolates originating from Norway spruce and Scots pine, respectively.

The datasets of IVGR and SP were reduced to pivot tables (Bimonte *et al.* 2021) by averaging the values of IVGR and SP for each isolate. The corresponding pivot tables were merged (Crawley 2013) based on the common field reporting the isolate codes. The correlation between *in vitro* mycelial growth rate and sporulation was tested on the merged dataset by calculating the Spearman's Rho coefficient along with its corresponding *P*-value (Crawley 2013). The overall trade-off detected between growth rate and sporulation *in vitro* (see results) was appraised from the same dataset by calculating the incremental change $\frac{\Delta\, million\ spores/_{plate}}{\Delta\, mm\ micelial\ growth/_{day}}$ as slope coefficient (m) of the linear equation interpolating the IVGR and SP values, displayed as point features in a scatterplot. The *P*-value of the t statistics associated with m was obtained as reported in Crawley (2013). A visual representation of the trade-off between IVGR and SP was obtained by fitting a standard deviational ellipse (Mitchell 2009) to the point features coordinates of the above scatterplot. The ellipse standard deviation parameter was set at 0.5 standard deviation units (Mitchell 2009). To test for significant thresholds of IVGR associated with significant reductions in SP ($P<0.05$), an unbiased recursive partitioning tree model was fitted on SP (response variable) and IVGR (input continuous variable) as previously described (Hothorn *et al.* 2006, Hothorn and Zeileis 2015). The same Spearman's

correlation analysis and the fitting of the standard deviational ellipse were performed separately on points related to isolates from Norway spruce and Scots pine.

Wood growth rate (WGR) was included as response variable to fit unbiased recursive partitioning conditional inference-based trees and random forests models (Hothorn *et al.* 2006, Strobl *et al.* 2008, 2009, Hothorn and Zeileis 2015). Models were fitted on the whole set of available data and on a subset. The whole set included the WGR of isolates of *P. gigantea* obtained from Norway spruce and inoculated in wood samples of Norway spruce (41 isolates, 1039 replicates) or Scots pine (17 isolates, 374 replicates), and isolates from Scots pine inoculated in Scots pine (15 isolates, 326 replicates) or Norway spruce (23 isolates, 443 replicates). The subset included 15 isolates obtained from Norway spruce and inoculated in logs of Norway spruce (270 replicates) and Scots pine (334 replicates), and 14 isolates obtained from Scots pine and inoculated in logs of Norway spruce (254 replicates) and Scots pine (304 replicates). Tree models were fitted as described above, by including as predictors either the host of origin of the isolates, or the wood species in which the isolates were inoculated, or both. Terminal node averages displayed by WGR were contrasted based on the resulting splits at $P<0.05$ (Hothorn *et al.* 2006, Hothorn and Zeileis 2015), while models' comparison was carried out as described in Lione *et al*. 2020 by calculating the Theil's UII forecast accuracy coefficient (Bliemel 1973). Random forests were fitted to assess whether, and to what extent, the growth rate in wood of isolates may depend upon the host they originate from, or upon the host they colonize. Random forests iteration process was based on the two-predictor tree model described above for WGR, by setting the algorithm with the default parameters (Strobl *et al.* 2009, Hothorn and Zeileis 2015). For each predictor (i.e., host of origin of the isolates and the wood species in which they were inoculated), the strength of its causal effect on the growth rate of isolates in wood was quantified by calculating the variable importance as reported in Strobl *et al.* (2009).

For comparative purposes, variables related to growth rates and sporulation were calculated also for the Rotstop® strain of *P. gigantea* (12 replicates for IVGR, 10 for SP, 196 for WGR on Norway spruce and 112 on Scots pine), yet the above values were excluded from the overall statistical analyses.

All averages were calculated along with the 95% bias-corrected and accelerated (BCa) confidence intervals ($CI_{95\%}$) (DiCiccio and Efron 1996) by setting the number of bootstrap iterations as reported in Lione *et al.* (2021).

Data processing and statistical analyses were conducted with R version 3.6.0 (R Core Team, 2019) and with the packages bootstrap (Efron and Tibshirani 1994), *party* and *partykit* (Hothorn and Zeileis 2015), *pivottabler* (Bailiss 2021), and *phonTools* (Barreda 2015). The significance threshold was set to 0.05 for all analyses (Crawley 2013).

References

Bailiss C., (2021). pivottabler: Create Pivot Tables. R package version 1.5.3. https://CRAN.R-project.org/package=pivottabler

Barreda, S. (2015). phonTools: Functions for phonetics in R. *R package version 0.2-2.1*. https://CRAN.R-project.org/package=phonTools

Bimonte, S., Antonelli, L., & Rizzi, S. (2021). Requirements-driven data warehouse design based on enhanced pivot tables. *Requirements Engineering*, *26*(1), 43-65.

Bliemel, F. (1973). Theil's forecast accuracy coefficient: A clarification. *Journal of Marketing Research, 10*, 444-446.

Crawley MJ (2013) The R Book, 2nd ed. John Wiley and Sons

DiCiccio, T. J., & Efron, B. (1996). Bootstrap confidence intervals. *Statistical science*, *11*(3), 189-228.

Efron, B., and Tibshirani, R. J. 1994. An introduction to the bootstrap. R package version 2019.6. https://cran.r-project.org/web/packages/bootstrap/index.html

Hothorn, T., Hornik, K., & Zeileis, A. (2006). Unbiased recursive partitioning: A conditional inference framework. *Journal of Computational and Graphical statistics*, *15*(3), 651-674.

Hothorn, T., Zeileis, A. (2015). partykit: A modular toolkit for recursive partytioning in R. *Journal of Machine Learning Research*, *16*, 3905-3909.

Lione, G., Giordano, L., Turina, M., & Gonthier, P. (2020). Hail-induced infections of the chestnut blight pathogen *Cryphonectria parasitica* depend on wound size and may lead to severe diebacks. *Phytopathology*, *110*(7), 1280-1293.

Lione, G., Giordano, L., Sillo, F., Brescia, F., & Gonthier, P. (2021). Temporal and spatial propagule deposition patterns of the emerging fungal pathogen of chestnut *Gnomoniopsis castaneae* in orchards of north-western Italy. *Plant Pathology*, *70*(9), 2016-2033.

Mitchell, A. (2009) The ESRI Guide to GIS Analysis – Volume 2 – Spatial Measurement and Statistics. ESRI Press.

R Core Team. 2019. R: A language and environment for statistical computing. R Foundation for Statistical Computing, Vienna, Austria. https://www.R-project.org/ Google Scholar

Strobl, C., Boulesteix, A. L., Kneib, T., Augustin, T., & Zeileis, A. (2008). Conditional variable importance for random forests. *BMC bioinformatics*, *9*(1), 1-11.

Strobl, C., Malley, J., & Tutz, G. (2009). An introduction to recursive partitioning: rationale, application, and characteristics of classification and regression trees, bagging, and random forests. *Psychological methods*, *14*(4), 323.
