# Supplementary material for: Host-associated Intraspecific Phenotypic Variation in the Saprobic Fungus Phlebiopsis gigantea
Source: Microb Ecol. 2023 Jan 28;86(3):1847–55. doi: 10.1007/s00248-023-02176-z (PMC10497652; doi:10.1007/s00248-023-02176-z)
Supplement: Supplementary file 3 — Supplementary file3. Online Resource 3 Clustering of the isolates of Phlebiopsis gigantea based on their in vitro growth rate (PDF 576 KB) [file 248_2023_2176_MOESM3_ESM.pdf]

MICROBIAL ECOLOGY

Host-associated intraspecific phenotypic variation in the saprobic fungus *Phlebiopsis gigantea*

Dārta Kļaviņa [1], Guglielmo Lione [2*], Kristīne Kenigsvalde [1], Martina Pellicciaro [2], Indriķis Muižnieks [3], Lauma Silbauma [1], Jurģis Jansons [1], Tālis Gaitnieks [1] and Paolo Gonthier [2]

[1] Latvian State Forest Research Institute Silava, Rigas street 111, LV-2169, Salaspils, Latvia.

[2] Department of Agricultural, Forest and Food Sciences (DISAFA), University of Torino, Largo Paolo Braccini 2, I-10095, Grugliasco, Italy.

[3] Department of Microbiology and Biotechnology, University of Latvia, Jelgavas street 1, LV- 1586, Riga, Latvia.

*Corresponding author: Guglielmo Lione (email: guglielmo.lione@unito.it)

ONLINE RESOURCE 3

Clustering of the isolates of *Phlebiopsis gigantea* based on their *in vitro* growth rate (mm/day)

For each cluster (on the *x*-axis) the corresponding average growth rate is reported (*y*-axis) with the related lower and upper bounds of the 95% confidence interval. Different letters mark significant differences (P<0.05).

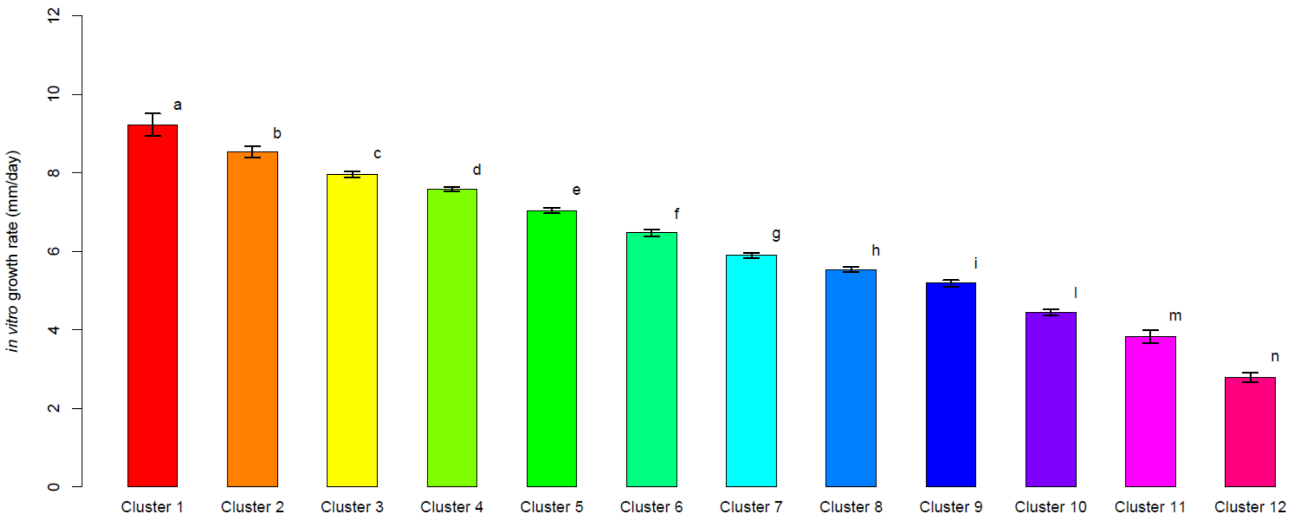

The list of isolates included within each cluster in reported in the tables below as follows: Table A reports the list of isolates in the clusters. Table B shows the number of isolates from Norway spruce and Scots pine embedded within each cluster, while Table C reports the same information expressed in percent (%) on the total number of isolates listed within the cluster.

Table A

	Cluster 1	Cluster 2	Cluster 3	Cluster 4	Cluster 5	Cluster 6	Cluster 7	Cluster 8	Cluster 9	Cluster 10	Cluster 11	Cluster 12
**Isolates**	C2	E107P	C1	D107P	Gi307P	B707E	Ap0111P	B507E	J207P	In208E	Kn207P	Z1111E
	NA0210E	Gi107P	J3	J1	In108P	J1007P	B107E	Gi207P	J907P	J1607P	Z0111E	Z1211E
	G20110P	J2	J4	K1	J107P	J307P	B207E	J1707P	K107P	K0111E	Z0411E	Z1411E
		K4	K2	Kd208P	J1107P	Kd207P	B307E	Kd308E	Kd108P	K0211E	Z0911E	
		NC0110E	K207P	Le207P	J1207P	Kd408P	B407E	Og0111E	Og0211E	K108P		
		G20510P	K3	Le407P	J1307P	Kd608P	B607E	Og0311E	Ti208E	S107P		
			Kd1	Le707P	J1407P	Kn107E	Ba0111P		Z0211E	Ti408E		
			Kd107E	O1	J1507P	Le507P	J407P		Z0511E	Ti508E		
			Kd2	O107E	J507P	M108E	J807P		Z1511E	Z0611E		
			Kd3	O2	J607P	N107P	K307P			Z0711E		
			Le307P	Pl	J707P	O208E	Le107E			Z0811E		
			O207E	Ti108E	K208P	S207P	Le607P			Z1011E		
			Sk107E	Ti608E	K407P	T207E	N207P			Z1311E		
			G20210P	NC0210E	Kd508P	NA0110E	O108E					
				G20310P	Kn1		Og0411E					
					L108P		Z0311E					
					Le807P		G20810P					
					M208E							
					T107E							

Table B

	Cluster 1	Cluster 2	Cluster 3	Cluster 4	Cluster 5	Cluster 6	Cluster 7	Cluster 8	Cluster 9	Cluster 10	Cluster 11	Cluster 12	Total
**Isolates from Norway spruce**	1	2	5	8	2	6	9	4	5	10	3	3	58
**Isolates from Scots pine**	2	4	9	7	17	8	8	2	4	3	1	0	65
**Total**	3	6	14	15	19	14	17	6	9	13	4	3	123

Table C

	Cluster 1	Cluster 2	Cluster 3	Cluster 4	Cluster 5	Cluster 6	Cluster 7	Cluster 8	Cluster 9	Cluster 10	Cluster 11	Cluster 12
**Isolates from Norway spruce**	33%	33%	36%	53%	11%	43%	53%	67%	56%	77%	75%	100%
**Isolates from Scots pine**	67%	67%	64%	47%	89%	57%	47%	33%	44%	23%	25%	0%
