# Supplementary material for: Host-associated Intraspecific Phenotypic Variation in the Saprobic Fungus Phlebiopsis gigantea
Source: Microb Ecol. 2023 Jan 28;86(3):1847–55. doi: 10.1007/s00248-023-02176-z (PMC10497652; doi:10.1007/s00248-023-02176-z)
Supplement: Supplementary file 4 — Supplementary file4. Online Resource 4 Clustering of the isolates of Phlebiopsis gigantea based on their in vitro sporulation (PDF 565 KB) [file 248_2023_2176_MOESM4_ESM.pdf]

MICROBIAL ECOLOGY

Host-associated intraspecific phenotypic variation in the saprobic fungus *Phlebiopsis gigantea*

Dārta Kļaviņa [1], Guglielmo Lione [2*], Kristīne Kenigsvalde [1], Martina Pellicciaro [2], Indriķis Muižnieks [3], Lauma Silbauma [1], Jurģis Jansons [1], Tālis Gaitnieks [1] and Paolo Gonthier [2]

[1] Latvian State Forest Research Institute Silava, Rigas street 111, LV-2169, Salaspils, Latvia.

[2] Department of Agricultural, Forest and Food Sciences (DISAFA), University of Torino, Largo Paolo Braccini 2, I-10095, Grugliasco, Italy.

[3] Department of Microbiology and Biotechnology, University of Latvia, Jelgavas street 1, LV- 1586, Riga, Latvia.

*Corresponding author: Guglielmo Lione (email: guglielmo.lione@unito.it)

ONLINE RESOURCE 4

Isolates of *Phlebiopsis gigantea* included in each cluster identified based on *in vitro* sporulation (million/plate)

For each cluster (on the *x*-axis) the corresponding average sporulation is reported (*y*-axis) with the related lower and upper bounds of the 95% confidence interval. Different letters mark significant differences ($P<0.05$).

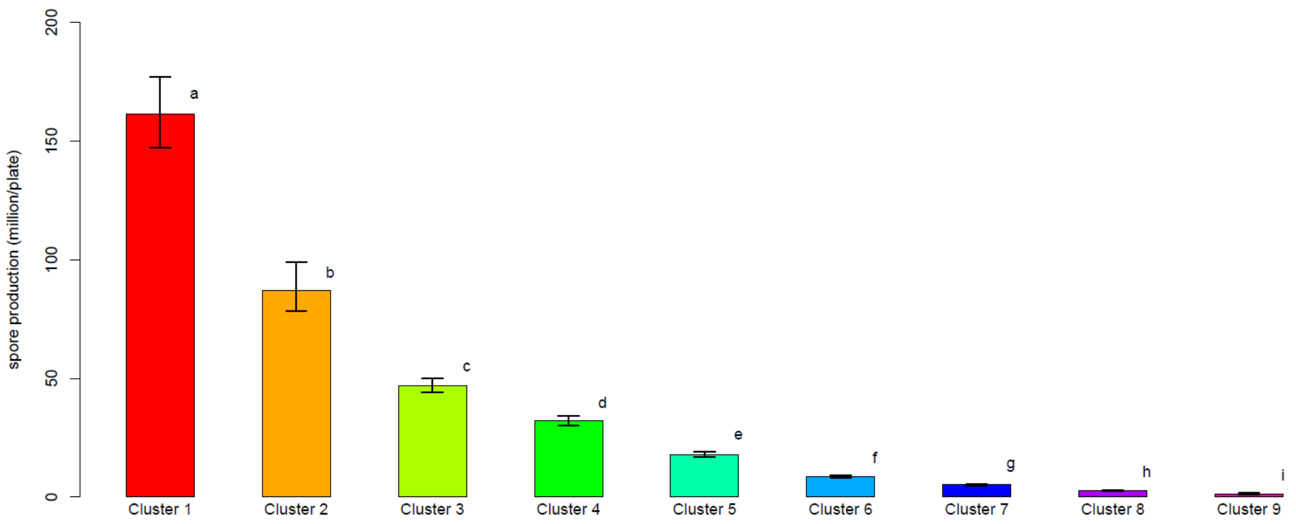

The list of isolates included within each cluster in reported in the tables below as follows: Table A reports the list of isolates in the clusters. Table B shows the number of isolates from Norway spruce and Scots pine embedded within each cluster, while Table C reports the same information expressed in percent (%) on the total number of isolates listed within the cluster.

Table A

Cluster 1	Cluster 2	Cluster 3	Cluster 4	Cluster 5	Cluster 6	Cluster 7	Cluster 8	Cluster 9
Z0711E	J2	Gi107P	Ba0111P	B507E	B607E	Gi207P	K207P	G20810P
Z0911E	J3	Gi307P	C2	In208E	B707E	G20210P	Kd107E	J1507P
Z1111E	K0211E	In108P	D107P	J1	G20110P	J507P	Le807P	Ti108E
Z1411E	Kd408P	L108P	J207P	J1007P	G20310P	J907P	Og0411E	
	Z0111E	O108E	J607P	J107P	G20510P	K3	Pl	
	Z0611E	O208E	J707P	J1107P	J1207P	Kd207P		
	Z1011E	Og0111E	K0111E	J1307P	J1607P	Kd508P		
	Z1211E	Og0211E	K4	J1407P	J307P	NA0210E		
	Z1311E	Og0311E	Kd1	J1707P	K108P	T107E		
	Z1511E	Z0811E	Kd108P	J4	K2	Ti508E		
			Kd2	J407P	K208P	Z0411E		
			Le207P	J807P	Kd208P			
			O207E	K107P	Kd3			
			Ti408E	K307P	Kn207P			
			Z0211E	K407P	Le307P			
			Z0511E	Kd308E	M108E			
				Kd608P	M208E			
				Kn1	NA0110E			
				Kn107E	NC0110E			
				Le107E	O1			
				Le407P	S107P			
				Le707P	Ti608E			
				N107P	Z0311E			
				N207P				
				NC0210E				
				O107E				

	Cluster 1	Cluster 2	Cluster 3	Cluster 4	Cluster 5	Cluster 6	Cluster 7	Cluster 8	Cluster 9
					O2				
					O107E				
					S207P				

Table B

	Cluster 1	Cluster 2	Cluster 3	Cluster 4	Cluster 5	Cluster 6	Cluster 7	Cluster 8	Cluster 9	Total
**Isolates from Norway spruce**	5	7	6	7	9	13	5	4	1	57
**Isolates from Scots pine**	0	4	5	10	22	13	7	2	3	66
**Total**	5	11	11	17	31	26	12	6	4	123

Table C

	Cluster 1	Cluster 2	Cluster 3	Cluster 4	Cluster 5	Cluster 6	Cluster 7	Cluster 8	Cluster 9
**Isolates from Norway spruce**	100%	64%	55%	41%	29%	50%	42%	67%	25%
**Isolates from Scots pine**	0%	36%	45%	59%	71%	50%	58%	33%	75%
