# Supplementary material for: Host-associated Intraspecific Phenotypic Variation in the Saprobic Fungus Phlebiopsis gigantea
Source: Microb Ecol. 2023 Jan 28;86(3):1847–55. doi: 10.1007/s00248-023-02176-z (PMC10497652; doi:10.1007/s00248-023-02176-z)
Supplement: Supplementary file 5 — Supplementary file5. Online Resource 5 Comparison of growth rates (mm/day) of Phlebiopsis gigantea isolates from Norway spruce and Scots pine inoculated in wood samples of both host species referring to the data subset (PDF 512 KB) [file 248_2023_2176_MOESM5_ESM.pdf]

MICROBIAL ECOLOGY

Host-associated intraspecific phenotypic variation in the saprobic fungus *Phlebiopsis gigantea*

Dārta Kļaviņa [1], Guglielmo Lione [2]*, Kristīne Kenigsvalde [1], Martina Pellicciaro [2], Indriķis Muižnieks [3], Lauma Silbauma [1], Jurģis Jansons [1], Tālis Gaitnieks [1] and Paolo Gonthier [2]

[1] Latvian State Forest Research Institute Silava, Rigas street 111, LV-2169, Salaspils, Latvia.

[2] Department of Agricultural, Forest and Food Sciences (DISAFA), University of Torino, Largo Paolo Braccini 2, I-10095, Grugliasco, Italy.

[3] Department of Microbiology and Biotechnology, University of Latvia, Jelgavas street 1, LV- 1586, Riga, Latvia.

*Corresponding author: Guglielmo Lione (email: guglielmo.lione@unito.it)

ONLINE RESOURCE 5

Comparison of growth rates of *Phlebiopsis gigantea* isolates from Norway spruce and Scots pine inoculated in wood samples of both host species referring to the data subset

Comparison of growth rates (mm/day) of *Phlebiopsis gigantea* isolates from Norway spruce (A) and Scots pine (B) inoculated in wood samples of both host species referring to the data subset. The data subset includes 15 isolates obtained from Norway spruce and inoculated in logs of Norway spruce and Scots pine, and 14 isolates obtained from Scots pine and inoculated in logs of Norway spruce and Scots pine. Error bars refer to the lower and upper bounds of the 95% confidence interval. Different letters mark significant differences ($P<0.05$).

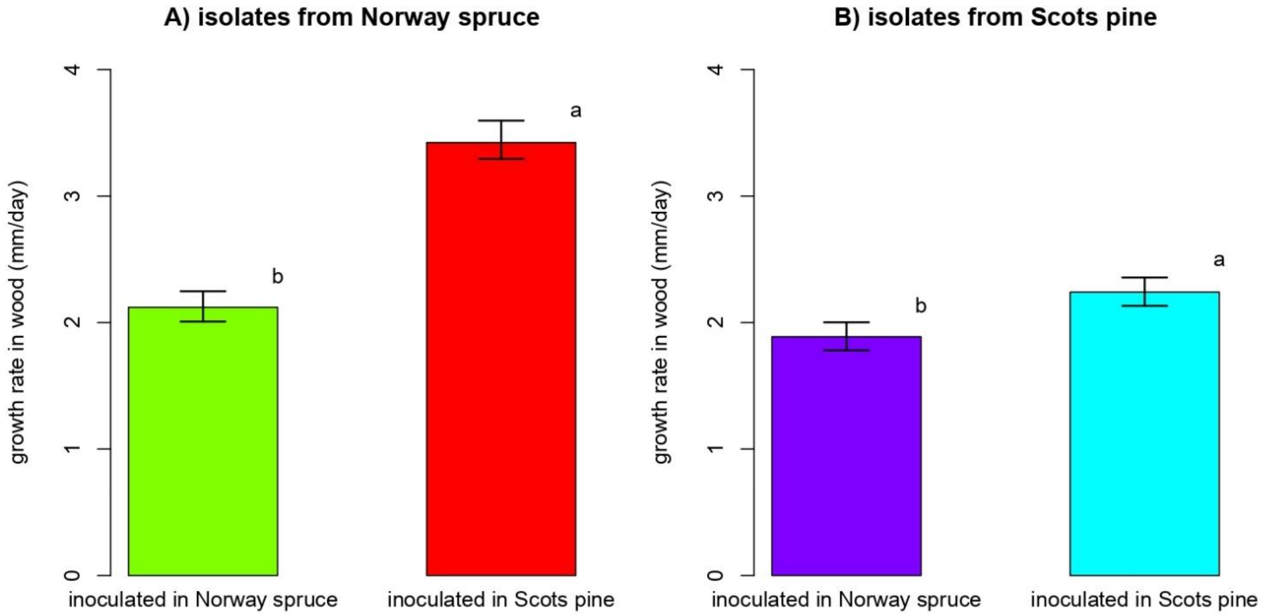

The analysis of the data subset showed in the figure above showed an average WGR of 3.42 mm/day (3.29-3.59 mm/day $CI_{95\%}$) displayed by isolates of Norway spruce inoculated in Scots pine, 2.24 mm/day (2.13-2.36 mm/day $CI_{95\%}$) by isolates from Scots pine and inoculated in Scots pine, 2.12 mm/day (2.01-2.25 mm/day $CI_{95\%}$) by isolates from Norway spruce inoculated in Norway spruce, and 1.89 mm/day (1.78-2.00 mm/day $CI_{95\%}$) by isolates from Scots pine inoculated in Norway spruce.
